# Resistive switching in optoelectronic III-V materials based on deep traps

**DOI:** 10.1038/s41598-018-27835-x

**Published:** 2018-06-21

**Authors:** M. Schnedler, V. Portz, U. Semmler, M. Moors, R. Waser, R. E. Dunin-Borkowski, Ph. Ebert

**Affiliations:** 1 Peter Grünberg Institut, Forschungszentrum Jülich GmbH, Jülich, 52425 Germany; 2 Institut für Werkstoffe der Elektrotechnik II (IWE II), RWTH Aachen University, Aachen, 52074 Germany; 3 Ernst Ruska-Centrum, Forschungszentrum Jülich GmbH, Jülich, 52425 Germany

## Abstract

Resistive switching random access memories (ReRAM) are promising candidates for energy efficient, fast, and non-volatile universal memories that unite the advantages of RAM and hard drives. Unfortunately, the current ReRAM materials are incompatible with optical interconnects and wires. Optical signal transmission is, however, inevitable for next generation memories in order to overcome the capacity-bandwidth trade-off. Thus, we present here a proof-of-concept of a new type of resistive switching realized in III-V semiconductors, which meet all requirements for the implementation of optoelectronic circuits. This resistive switching effect is based on controlling the spatial positions of vacancy-induced deep traps by stimulated migration, opening and closing a conduction channel through a semi-insulating compensated surface layer. The mechanism is widely applicable to opto-electronically usable III-V compound semiconductors.

## Introduction

Resistive random access memories (ReRAMs) were recently shown to match conventional dynamic random access memories’ (DRAMs) latency ratings and readout performance^1^. ReRAM as an successor of DRAM would entail numerous advantages like fast, non-volatile, and energy efficient^2,3^ data storage and access. However, the latency, i.e. the time interval between the processor’s request and the memories’ response, and signal degradation caused by on-chip wires and transmission lines are bottlenecks of today’s DRAM performance^4,5^, which will be inherited by next generation ReRAMs. To overcome these bottlenecks, fast optical interconnections in ReRAM arrays are becoming inevitable. However, the implementation of integrated photonic- and electronic circuits on a single chip is highly challenging due to manufacturing conflicts and the lack of suitable material systems^6^. Therefore, we present a new type resistive switching effect in a III-V semiconductor with excellent prospects for integrating resistive switching with photonic circuits.

In contrast to present DRAMs and flash memories, which store information by electric charging, ReRAMs are based on the reversible switching of the resistivity of a suitable metal-insulator-metal system between a low-resistive state (LRS) and a high-resistive state (HRS). Today, many competing concepts for future ReRAMs exist. These are categorized either phenomenologically or according to the underlying physical effect^3,7,8^: The most prominent physical effects are atomic structure changes (e.g. phase changes^9,10^ or molecular switching), as well as redox reactions combined with ion motion (e.g. electrochemical metallization or valency change effects)^11–17^. However, typical resistive switching materials, such as, chalcogenides, (transition) metal oxides, and complex oxides, are incompatible with optoelectronic III-V materials. The discovery of resistive switching in photonic material systems would open the path to novel ReRAMs by combining advantages of fast optical information transfer with resistive information storage.

Here we present a new type of filamentary reversible resistive switching effect in InP, a prototype optoelectronic III-V material. It is based on switching the resistivity of a subsurface hole injection blocking layer by controlled motion of vacancy-induced deep traps. The high-to-low resistive switching mechanism is found to be a local field-driven repulsive vacancy motion creating a deep trap-free channel through the hole injection blocking layer. Reversibility is achieved by thermal back switching without an applied electric field, refilling the channel with deep traps. This new deep trap motion-thermal switching mechanism is expected to occur in other optoelectronic III-V compound semiconductors, too.

For demonstrating resistive switching in InP, we utilize the dependence of the conductivity on the concentration of deep traps associated with anion vacancies: At non-polar surfaces of *p*-type InP, Langmuir desorption of phosphorus occurs already at low temperatures (starting at 20 °C up to 270 °C)^18–20^ leading to numerous positively charged phosphorus ${{\rm{V}}}_{{\rm{P}}}^{+}$ vacancies (surface concentrations up to 6 × 10^12^ cm^−2^)^18,21^. Due to the small activation energy for vacancy in-diffusion^22^, the redistribution of vacancies is possible either by annealing or by applying electric fields. Hence, the precise control of the spatial distribution of ${{\rm{V}}}_{{\rm{P}}}^{+}$-induced deep traps in InP is the key for creating either a high or a low resistive state.

## Results

**Figure 1:**
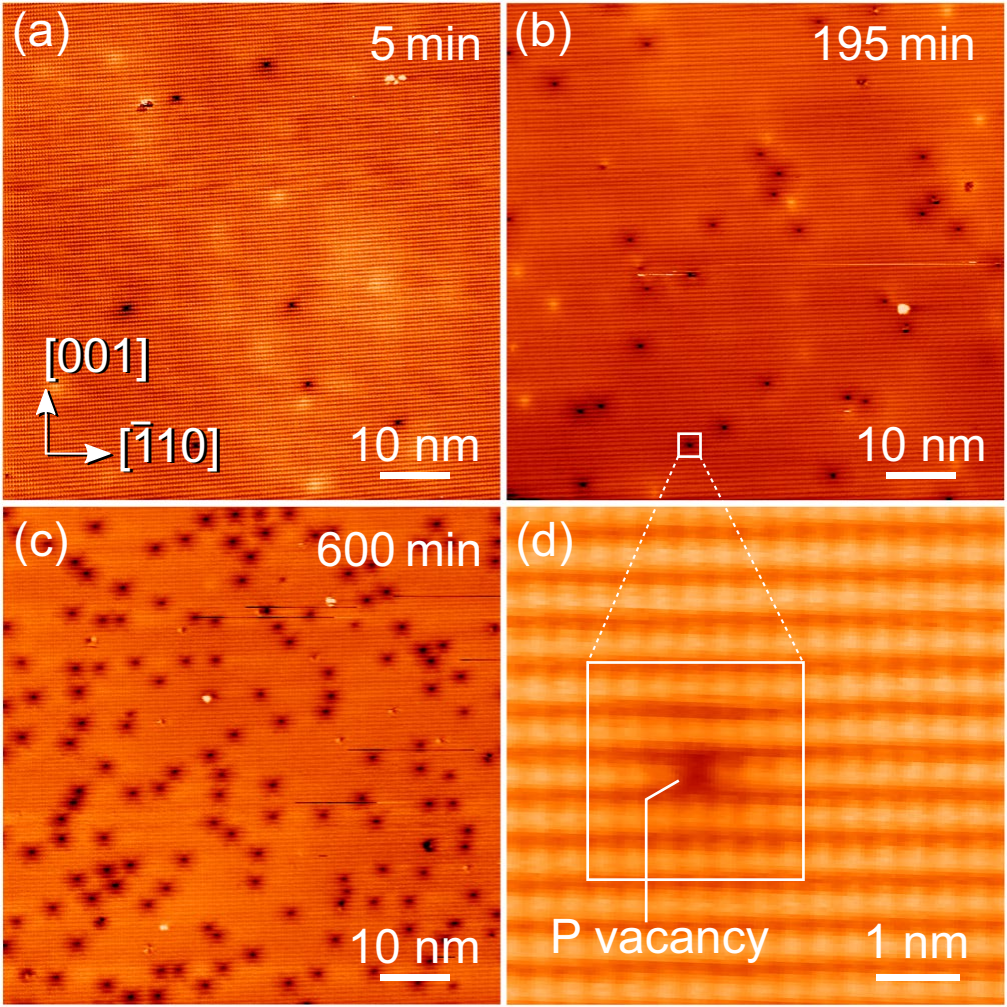
Filled state constant-current STM images of the *p*-doped InP(110) surface subjected to annealing at 120 °C for (**a**) 5, (**b**) 195, and (**c**) 600 min. The annealing creates positively charged ${{\rm{V}}}_{{\rm{P}}}^{+}$ vacancies showing up as single missing filled dangling bonds [see magnified STM image in (**d**)] surrounded by a depression due to the charge-induced screened Coulomb potential. The latter is primarily visible in the overview STM images (**a–c**). The ${{\rm{V}}}_{{\rm{P}}}^{+}$ vacancy concentration increases with annealing time.

First we turn to the preparation of the hole injection blocking layer. Figure 1(a–c) present overview constant-current scanning tunneling microscopy (STM) images of a freshly cleaved InP(110) surface subjected to total annealing times of 5, 195, and 600 min, respectively, at 120 °C. The STM images were acquired, after cooling to room temperature between each annealing period, using negative sample voltages. Hence, the phosphorus sublattice (filled dangling bond states) of the InP(110) surface from wafer (i) (cf. Methods section) has been imaged. In these images, ${{\rm{V}}}_{{\rm{P}}}^{+}$ vacancies show up as single missing filled dangling bonds [visible in the atomically resolved STM image in Fig. 1(d)], which are surrounded by 4–5 nm wide apparent depressions^23^. The depressions arise from local downward band bendings induced by the positive vacancy charges^24,25^. The vacancy concentrations increase with annealing time and vacancies are formed by Langmuir desorption of phosphorus^18,26^.

**Figure 2:**
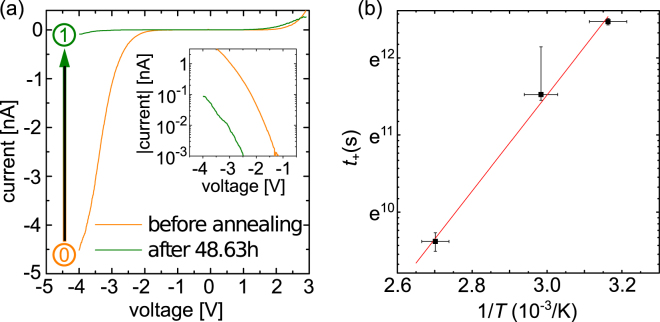
(**a**) Current-voltage tunnel characteristics of the InP(110) surface before (orange curve labeled 0) and after (green curve labeled 1) annealing to 90 °C for 48.64 h acquired at a set-point of +3.0 V and 300 pA. The tunnel current at negative sample voltages is reduced by a factor of $\sim 50$ after annealing. Inset: logarithmic display of the tunnel currents at negative voltages highlighting the different current onsets. (**b**) Time between the start of the annealing process and the collapse of tunneling at moderate negative sample voltages, *t*_off_, as a function of the inverse annealing temperature. The logarithmic scale demonstrates an Arrhenius behavior with a diffusion barrier of (0.54 ± 0.14) eV.

We now turn to the question, if and how this rather high concentration of vacancies present at the InP(110) surface influences the conductivity. *I*-*V* characteristics were probed by scanning tunneling spectroscopy (STS) on cleavage surfaces of wafer (ii) (cf. Methods section), annealed constantly to either 40 °C, 60 °C, or 90 °C. While these temperatures were kept constant, *I*-*V* characteristics were measured in regular intervals, but constantly changing lateral positions on the hot sample surface, to avoid any effect of the tip’s electric field during probing. Figure 2(a) illustrates *I*-*V* characteristics before (orange curve labeled 0) and after (green curve labeled 1) annealing the cleavage surfaces to 90 °C for 48.64 h. We found, independent of the tip used, a decreasing tunnel current at negative sample voltages with annealing time and thus with increasing vacancy concentrations.

**Figure 3:**
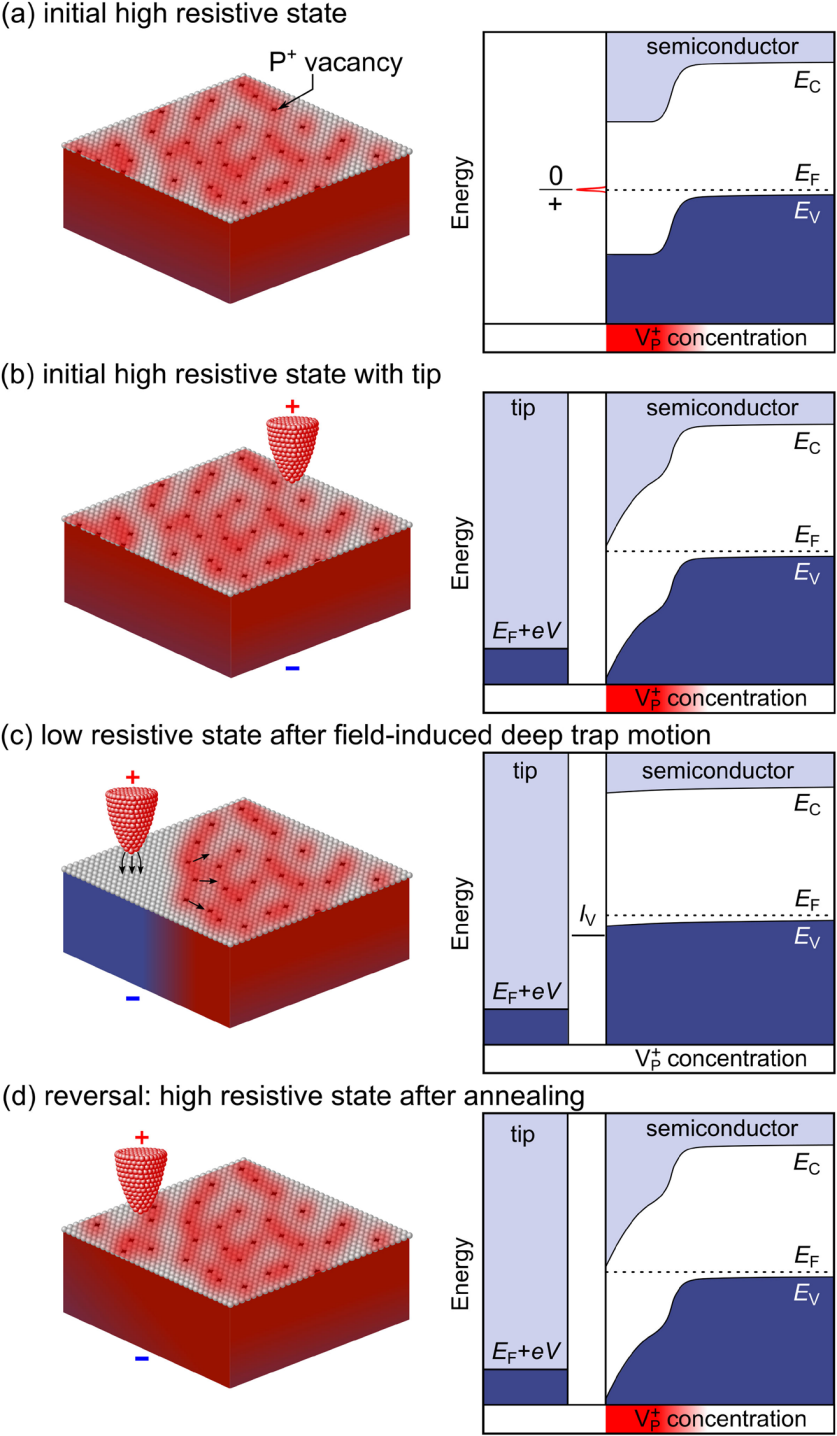
Schematics of the different resistive states with respective band diagrams. (**a**) Without tip, ${{\rm{V}}}_{{\rm{P}}}^{+}$ vacancies diffuse from the surface towards the bulk and compensate the Zn/Cd dopants. As a consequence, under equilibrium conditions, the free carrier concentration is reduced tremendously in this region, leading to mid-gap Fermi level pinning at the +/0 charge transition level of the ${{\rm{V}}}_{{\rm{P}}}^{+}$ vacancies. (**b**) Approaching a positively biased tip (i.e. negative sample bias) induces a strong downward band bending at the semiconductor surface. This so-called tip-induced band bending (TIBB) ultimately shifts the valence band edge below the tip’s Fermi energy, blocking electron tunneling and thus forming a hole injection blocking layer. Thus, no tunnel current is measured at negative voltages forming a high resistive state (HRS). (**c**) The HRS can be switched to the low-resistive state (LRS) by applying a −10 V pulse to the sample, which induces a field-driven repulsive motion of ${{\rm{V}}}_{{\rm{P}}}^{+}$ vacancies forming a vacancy free conductive channel. Within this channel, the increased concentration of free carriers screens the electrostatic field between the tip and the sample well enough to reduce the TIBB, remove the hole injection blocking layer, and thus increase the tunnel current. (**d**) The LRS can be switched back to the HRS by annealing, because ${{\rm{V}}}_{{\rm{P}}}^{+}$ diffusion closes the conductive channel.

The decreasing tunnel current is related to a shift of the onset voltages: Before annealing, the pristine surface exhibits small onset voltages of the tunnel current close to 0 V [cf. orange spectrum (0) in Fig. 2(a)], due to a sufficiently high concentration of free carriers screening the tip-induced band bending [see analogous band diagram in Fig. 3(c)]^27^. After annealing, the current onset is shifted to larger negative voltages of < −2.0 V [cf. green spectrum (1) in Fig. 2(a)]. In the STM images this effect shows up by increasing instabilities of the tunnel current and, after a certain time, the complete loss of resolution at negative sample voltages. The shift of the onset voltages can be explained by in-diffusion of vacancy-induced deep traps from the surface into the subsurface layers, which has been shown to occur in this temperature range^21,22^. The positively charged subsurface vacancies compensate the ionized, negatively charged *p*-type dopants^21,22^. Hence, the free carrier concentration is strongly reduced and the Fermi level moves towards a mid-gap position near the surface. The sketch of the InP(110) surface and the corresponding band bending diagram (i.e. graph of the semiconductor’s conduction- and valence band edge as a function of the distance from the semiconductor’s surface) is shown in Fig. 3(a). Note, in thermal equilibrium and without the presence of a probe tip, the rather high vacancy concentration at the surface additionally pins the Fermi level at the +/0 charge transition level in mid-gap position, approximately 0.75 eV above the valence band edge *E*_V_^28^. However, this Fermi level pinning has no effect on our experiment, since it cannot be preserved under tunneling conditions [due to the lack of electrons required for the charge transition].

If a metallic probe tip is approached and a negative sample voltage is applied, as depicted in Fig. 3(b), a strong downward band bending is induced at the semiconductor surface. This so-called tip-induced band bending (TIBB) arises due to the low free carrier concentration insufficient to screen the electrostatic field between the tip and the sample^27,29^. Ultimately, when the vacancy in-diffusion compensated a wide enough subsurface layer, the TIBB shifts the valance band edge below the Fermi level of the tip [*E*_F,tip_ = *E*_F_ + *eV*, see Fig. 3(b)], blocking electron tunneling and hence hole injection into the valence band. Thus, a hole-injection blocking layer develops. Hence, we expect that the InP(110) layer with a subsurface vacancy-induced hole injection blocking layer exhibits an electrically insulating behavior at negative voltages in agreement with the observed disappearance of the tunnel current at moderate negative voltages. Note, at positive sample voltages a majority carrier accumulation is induced at the surface which screens the TIBB and thus tunneling is still possible.

The observed time *t*_off_ between the start of the annealing and the complete collapse of the tunnel current at moderate negative voltages depends on the annealing temperature, as illustrated by the Arrhenius plot in Fig. 2(b). A clear exponential dependence is found. For modeling this temperature dependence, we assume an in-diffusion of ${{\rm{V}}}_{{\rm{P}}}^{+}$ with a temperature dependent diffusion coefficient *D* = *D*_0_ × exp(−*E*_d_/*kT*). The depth of penetration^30^ of the vacancies *x*_off_ at the time *t*_off_, where the tunneling at negative voltages collapses, is then given by ${x}_{{\rm{off}}}=\sqrt{2D{t}_{{\rm{off}}}}$. Using these approximations, we obtain a diffusion barrier of *E*_d_ = (0.54 ± 0.14) eV from a linear fit to the data points [red line in Fig. 2(b)]. This energy is lower than that found previously in more bulk-like measurements^22,31^. This can be traced to the fact that the present measurement is based on near surface in-diffusion with small depths in the nm range and not, as previously, in the 0.1 mm range. Hence, the shape of the near surface electrostatic potential^32^ and repulsive Coulomb interactions between the charged surface vacancies^33,34^ have to be taken into account, lowering effectively the diffusion barrier. The low diffusion barrier supports in-diffusion and formation of a hole injection blocking layer which is active at moderate negative sample voltages.

**Figure 4:**
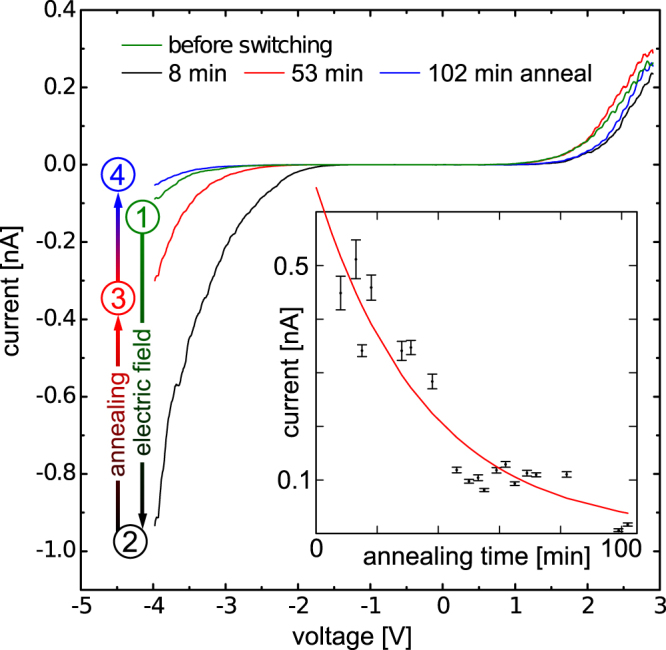
Current-voltage tunnel characteristics of the InP(110) surface (set-point: +3.0 V, 300 pA), annealed to 90 °C for different resistive states: The green curve labeled 1 corresponds to the high resistive state. The black *I*-*V* spectrum labeled 2 was obtained 8 min after switching to the low resistive state by applying a negative sample voltage of −10 V for ∼30 s. Continuous heating of the sample leads back to the initial high resistive state, as indicated by the red (53 min) and blue curve (102 min) labeled 3 and 4, respectively. Inset: Tunnel current at a sample voltage of −3.5 V as a function of the annealing time, illustrating the time dependence of the healing process of the hole injection blocking layer by annealing at 90 °C.

At this stage we define that the sample is in the HRS after annealing, when a homogeneous hole injection blocking layer has been created at and below the (110) surface due to the in-diffusion of ${{\rm{V}}}_{{\rm{P}}}^{+}$ vacancies. The definition of the HRS automatically raises the question, how we can achieve a LRS and how to reversibly switch back and forth between these two states? In order to answer this question, we turn to STS experiments performed on the InP(110) surface, which initially is in the HRS (annealed at 90 °C for 48.63 h). For switching from the HRS to LRS, a voltage pulse of −10 V was applied between the sample and the probe tip for approximately 30 s, corresponding to an electric field of $\sim 1\times {10}^{10}$ V/m (note, the actual value of the electric field within the surface region depends primarily on the tip-induced band bending). After the pulse, the tunnel current increases by a factor of 10 at negative voltages, while remaining unchanged at positive voltages, as shown by the black *I*-*V* characteristic (labeled 2) in Fig. 4 [compare to green curve (1), HRS].

This effect can be interpreted microscopically as follows: The −10 V switching pulse applies an extremely high local electrical field, which is known to induce a motion of positively charged vacancies away from the tip position, in accordance with the previously observed tip-induced (field-driven) vacancy migration under majority carrier injection^35–38^. As a result, a channel with reduced vacancy concentration and increased free carrier concentration forms through the hole injection blocking layer, as shown schematically in Fig. 3(c). The increased free carrier concentration leads to a reduced TIBB, comparable to that of an unpinned, vacancy-free InP(110) surface [cf. band banding diagram in Fig. 3(c)]. Hence, filled valance band states face again empty tip states and tunneling is possible, forming the LRS.

Interestingly, the LRS is present also far away from the local switched channel. STS reveals that tunneling is still possible at a distance of at least 14 *μ*m, indicating the presence of a surface conductivity. The surface conductivity can be provided by the A_5_ dangling bond surface state, since it is resonant with the valence band at the Γ-point and energetically situated directly at the valence band maximum^39^. Note, suppressing the surface conductivity through passivation layers eventually allows to create nanoscale resistive switches.

Finally, we focus on the process of switching back from the LRS (with a conductive channel being present) to the HRS. Figure 4 illustrates that further annealing (without the presence of the biased tip) leads to a time-dependent decrease of tunnel currents at negative sample voltages, as indicated by the black, red, and blue *I*-*V* characteristics labeled 2, 3, and 4. After annealing for 100 min, the *I*-*V* characteristic coincides with that measured before for the HRS (cf. blue and green *I*-*V* curve labeled 4 and 1 in Fig. 4). We have further studied the dependence of the annealing time on the tunnel current with higher time resolution, as depicted in the inset in Fig. 4. At a sample voltage of −3.5 V an exponential decay (decay constant of ∼36 min at 90 °C) of the tunnel current with increasing annealing time becomes visible. The exponential disappearance of the conductive channel indicates a diffusive healing of the hole injection blocking layer. The process of healing at elevated temperatures can be attributed to a concentration gradient and Coulomb repulsion driven diffusion of the positively charged vacancy-induced deep traps surrounding the conductive channel, reversing the field-induced diffusion used for switching from the HRS to the LRS. This mechanism of closure of the conductive channel is consistent with the low ${{\rm{V}}}_{{\rm{P}}}^{+}$ vacancy diffusion barrier measured.

## Discussion

We demonstrated a reversible switching between a low and a high resistive state of the InP(110) surface, governed by motion of vacancy-induced deep traps. The high resistive state is due to a hole injection blocking layer that is created by formation and in-diffusion of positively charged ${{\rm{V}}}_{{\rm{P}}}^{+}$ vacancies (and hence deep traps) in the subsurface layers. This hole injection blocking layer exhibits a tremendously low majority carrier concentration and thus rather bad screening properties. When biased by a probe tip and negative sample voltages, a large downward band banding prevents the emergence of a tunnel current. The low resistive state is triggered by applying a high electric field, which induces a repulsive motion of ${{\rm{V}}}_{{\rm{P}}}^{+}$ vacancies, locally depleting the vacancy concentration and creating a conducting channel through the hole injection blocking layer. The here found resistive switching effect is reversible, because subsequent annealing leads to a healing of the hole injection blocking layer by vacancy diffusion. However, following previous tip-induced vacancy migration effects, back-switching can be expected to be achieved also by reverse electric fields, leading to an attractive motion toward the tip position and thus refilling the conducting channel with ${{\rm{V}}}_{{\rm{P}}}^{+}$ vacancies, offering the prospect of greatly reduced switching times. The here found resistive switching mechanism differs fundamentally from the previously found ones based on the creation of conducting channels with a high concentration of shallow defects acting effectively as dopants. We propose to refer to this new filamentary switching mechanism as deep trap motion (DTM) switching mechanism. Similar DTM resistive switching can be expected to occur generally in III-V compound semiconductors, since their defect formation and deep trap energies are rather similar.

The proof of concept of resistive switching on III-V compound semiconductors, perfectly suited for photonic applications, offers the prospect for integrating ReRAM arrays with fast optical interconnects between them. Resistive switching memories based on optoelectronic materials would combine all advantages of ReRAMs, i.e. non-volatility and energy efficiency, with the advantages of optical information transmission, i.e. minimized signal degradation in on-chip wires and transmission lines, overcoming the capacitance-bandwidth trade-off of today’s memory modules.

## Methods

### Sample preparation

Samples cut from two commercially available *p*-doped InP wafers [(i): Zn (1.3–2.1) × 10^18^ cm^−3^, (ii) Cd 1.1 × 10^18^ cm^−3^ carrier concentration] were cleaved in ultrahigh vacuum at a pressure of 1 × 10^−10^ mbar. This resulted in clean and initially defect-free (110) surfaces, which were subjected to post-cleavage annealing. We estimate the temperature uncertainty to be 5 °C. The post-cleavage annealing initializes Langmuir evaporation of phosphorus, creating positively charged phosphorus vacancies acting as deep traps in the near surface region and creating a hole injection blocking  layer.

### STM measurements

Our experiments were carried out using variable temperature scanning tunneling microscopy (STM) and spectroscopy (STS) since this method offers the possibility to enhance vacancy generation and diffusion by either annealing or generating high electric fields between the probe tip and the sample. At the same time, it provides direct access to the local conductance. Hence, this method is ideally suited to set, reset, and probe (or read-out) the resistivity state. Furthermore, atomic scale defects can be directly identified in the atomically resolved STM images.

### Data availability

All relevant data are available from the authors upon reasonable request.
